# “It seems enormously valuable to me.” Perspectives of Dutch (potential) carriers of genetic FTD on onset-predictive biomarker testing

**DOI:** 10.1186/s13195-025-01749-z

**Published:** 2025-05-06

**Authors:** Charlotte H. Graafland, Harro Seelaar, Jessica L. Panman, Lize C. Jiskoot, Tjitske Kleefstra, Jackie M. Poos, Edo Richard, Maartje H.N. Schermer, John C. van Swieten, Laura Donker Kaat, Eline M. Bunnik

**Affiliations:** 1 Department of Public Health, section Medical Ethics, Philosophy and History of Medicine, Erasmus University Medical Centre, Rotterdam, The Netherlands; 2 Department of Neurology and Alzheimer Centre, Erasmus University Medical Centre, Rotterdam, The Netherlands; 3 Department of Clinical Genetics, Erasmus University Medical Centre, Rotterdam, The Netherlands; 4 Centre of Excellence for Neuropsychiatry, Vincent van Gogh Institute for Psychiatry, Venray, The Netherlands; 5 Department of Neurology, Donders Institute for Brain, Cognition and Behaviour, Radboud University Medical Centre, Nijmegen, The Netherlands; 6 Department of Public & Occupational Health, Amsterdam University Medical Centre, University of Amsterdam, Amsterdam, the Netherlands; 7 Erasmus School of Health Policy & Management, Erasmus University, Rotterdam, The Netherlands

**Keywords:** Onset prediction, Frontotemporal dementia, Biomarker, Ethics, Genetic counselling, Clinical trial recruitment, Stakeholder perspectives

## Abstract

**Background:**

Onset-predictive biomarker tests (OPBT) in genetic frontotemporal dementia (FTD) may be used to recruit mutation carriers into preventive clinical trials before symptoms manifest. This would require disclosure of OPBT results to potential participants. This study investigates the perspectives of Dutch presymptomatic mutation carriers and individuals at 50% risk of genetic FTD on disclosure of OPBT results. It focuses on their willingness to receive OPBT results, what impacts they foresee from disclosure, and their preferences for the process of disclosure.

**Methods:**

Semi-structured interviews were conducted with presymptomatic mutation carriers and individuals at 50% risk of developing genetic FTD (*n* = 25), who had received genetic counselling or participate in a longitudinal cohort study. The interview transcripts were analysed using thematic inductive analysis.

**Results:**

Main themes were: willingness to undergo biomarker testing, foreseen impact of test results, preferences regarding biomarker test features, and understanding of biomarker testing. Most participants would be willing to receive OPBT results in the context of clinical trial recruitment. Participants would also be willing to receive OPBT results without access to clinical trial participation, as they perceived utility from these results. They would use positive OPBT results to prepare for the future, e.g. by planning for care, drawing up advance care directives, retiring early, and spending final healthy years well. At the same time, they thought positive OPBT results might also have negative psychological impacts on self-image or social dynamics with others. Implications of positive OPBT results for self-image as healthy or ill differed between participants. Negative OPBT results would provide relief and not lead to life changes.

**Conclusions:**

Dutch presymptomatic mutation carriers and individuals at 50% risk of developing genetic FTD tend to be willing to receive OPBT results. The results would allow for participation in a clinical trial and preparation for onset through personal life planning. At the same time, disclosure of OPBT results might have negative psychological consequences. This study provides valuable input for developing ethical guidance and an appropriate counselling process to ensure responsible disclosure of OPBT results with clinical trial recruitment.

**Supplementary Information:**

The online version contains supplementary material available at 10.1186/s13195-025-01749-z.

## Background

Frontotemporal dementia (FTD) is typically characterised by early-onset behavioural changes, aphasia and/or motor dysfunction, with progressive atrophy predominantly in the frontal and temporal lobes [1–3]. FTD is familial in 30–40% of cases with heterozygous mutations in microtubule-associated protein tau (*MAPT)*, progranulin (*GRN)*, and chromosome 9 open reading frame 72 (*C9orf72)* genes as major causes [1]. Clinical trials with pharmacological interventions in genetic FTD are ongoing, mostly in symptomatic carriers. Yet, pathological brain changes occur long before clinical symptoms present. Therefore, new clinical trials will focus more and more on the presymptomatic or early symptomatic stage, in the hope that early treatment may prevent or delay symptom onset [1, 3–5].

In order to measure efficacy of experimental interventions within a reasonable time frame, trial participants should ideally have only mild symptoms or be expected to develop symptoms soon. However, it is difficult to predict symptom onset accurately based on genetic mutation alone, as age of symptom onset is highly variable even within families carrying the same mutation [1, 6]. Therefore, it is crucial to have biomarkers that reliably predict coming symptom onset in mutation carriers of FTD genes. A promising biomarker is neurofilament light chain (NfL), a non-specific marker of neuronal loss measured in blood or cerebrospinal fluid. Repeated measurements of serum NfL might be a suitable modality for monitoring disease activity in mutation carriers, as serum NfL is elevated in presymptomatic mutation carriers who will convert to the symptomatic stage within several years [7–11]. It could be combined with other biomarkers to predict onset accurately, with a ‘positive’ result indicating that symptom onset is expected soon. Presymptomatic mutation carriers of FTD genes with positive “onset-predictive biomarker testing” (OPBT) results could be invited to participate in clinical trials for FTD. Elevated serum NfL was recently used as an inclusion criterion to enrol presymptomatic *GRN* carriers in a clinical trial for latozinemab [12]. The result of the NfL test was disclosed to these mutation carriers following the principle of ‘transparent enrolment’, which states that prospective participants in clinical trials must be informed of the reason why they are invited to participate [13].

Disclosure of OPBT results to longitudinal cohort study participants may be ethically problematic. Almost half of at-risk participants in the longitudinal cohort study of FTD at Erasmus Medical Center, University Medical Centre Rotterdam, the Netherlands (Erasmus MC), do not wish to learn their genetic status (personal communication Lize Jiskoot, 5 December 2023). Most important reasons against presymptomatic genetic testing for neurodegenerative diseases in general are the absence of available treatments, worry about the psychological impact of testing positive, and the negative impact of this knowledge on life [14, 15]. The disclosure of OPBT results to cohort study participants in the context of clinical trial recruitment (i.e. ‘disclosure by enrolment’ [16]) may be problematic in those at 50% risk for FTD who have chosen not to learn their genetic status. They would learn both that they are mutation carriers and that they are expected to develop FTD in the coming years. This information may be harmful to them. Therefore, NfL testing has so far not been offered to individuals at 50% risk, only to known mutation carriers.

At this time, little is known about the willingness of presymptomatic mutation carriers and individuals at 50% risk to receive OPBT results. One study among participants in a longitudinal FTD study in the UK found that 85% of 135 survey participants would be willing to receive NfL results as part of a clinical trial [17]. Still, it remains unknown what exactly the perspectives of presymptomatic mutation carriers and individuals at 50% risk on OPBT are, what factors might influence willingness to receive OPBT results, and what impacts a positive result might have. Qualitative research is especially suitable to explore this knowledge gap. Its adaptable research design and interactions between researcher and participant allow for the collection of detailed and nuanced data. This article reports on qualitative interviews with known mutation carriers and individuals at 50% risk of developing FTD about their perspectives on OPBT. The results could be an important source of input for determining under which conditions and how OPBT results should be disclosed in the research context.

## Methods

### Sampling and recruitment

Semi-structured qualitative interviews were conducted with presymptomatic mutation carriers and individuals at 50% risk of carrying FTD (see Supplement 1 for an extensive description of methods). Most had no previous experience with OPBT (one participant had received negative NfL results). We used purposive sampling, aiming for diversity in age, education, and sex. Inclusion criteria were: (a) being ≥ 18 years old, (b) speaking Dutch, (c) carrying or being at 50% risk for carrying an identified pathogenic autosomal dominant mutation for FTD.

Participants were recruited from the Department of Clinical Genetics and from the longitudinal cohort study FTD-RisC, both at Erasmus MC. The FTD-RisC study aims to describe the natural history of genetic FTD by annually collecting data from known mutation carriers and individuals at 50% risk.

### Interview format

The interview guide was made by the FTD-RisC team (neurologists, clinical geneticists, neuropsychologists, medical ethicists). It included four topics: (1) family history of FTD, (2) considerations concerning presymptomatic genetic testing, (3) perspectives on OPBT, and (4) use of OPBT in clinical trials (for the full interview guide, see Supplement 2).

Interviews were conducted by Charlotte Graafland (CHG), an ethicist trained in qualitative methods, from September 2023 to August 2024 at the participant’s home or at Erasmus MC, depending on participant preferences. Participants provided written informed consent before the start of the interview. Interviews were audio-recorded. Recordings were transcribed verbatim using transcription software of Amberscript Global B.V. and manually checked for correctness by CHG. Transcripts were pseudonymised. CHG made personal notes detailing first impressions directly after each interview. Participants were invited until data saturation was reached, defined as finding no new information in the last three interviews [18].

### Data analysis

Thematic analysis of the interview transcripts was performed using NVivo R1(2020) software [19]. CHG and Eline Bunnik (EMB), an ethicist experienced in qualitative research, inductively constructed a codebook based on the first few transcripts. This codebook was adapted during further coding when necessary. All transcripts were coded separately by CHG and EMB, Jessica Panman (a neuropsychologist experienced with FTD) or another colleague of the section Medical Ethics, Philosophy and History of Medicine with knowledge on the topic. Coded transcripts were compared to determine the final coding of all transcripts and the final codebook. The study design and results are reported in accordance with COREQ guidelines (see Supplement 3) [20]. In the Results section, a ‘positive’ OPBT result refers to the prediction that symptom onset will occur soon, and a ‘negative’ result to the prediction that symptom onset is not expected yet.

## Results

### Participant characteristics

In total, 25 participants were included (9 from the Department of Clinical Genetics, 16 from the FTD-RisC study) from families with *C9orf72* (*n* = 17), *GRN* (*n* = 5), *MAPT* (*n* = 2) and *TAR DNA binding protein (TARDBP)* (*n* = 1). Two persons declined participation, one because they were busy, and one because they wanted to focus on other things than FTD at this time. Interviews had a duration of 32–92 min (average 64.4). The median age of participants was 44 years old (range 20–66) and all participants had Dutch nationality. 11 participants were known presymptomatic mutation carriers, while 14 participants were at 50% risk. Of individuals at 50% risk, 5 participants wanted to pursue presymptomatic genetic testing in the near future. Known mutation carriers had had genetic counselling, including consultation with a psychologist, before genetic testing and had received the genetic test result in the period between three weeks and fifteen years ago. An overview of the demographic details of participants is shown in Table 1.

**Table 1:** Demographic details of participants

	Total	Known carriers	At 50% risk
Number of participants	25	11	14
Sex			
Male Female	10 15	3 8	7 7
Age group			
20 to 29 years 30 to 39 years 40 to 49 years 50 to 59 years 60 to 69 years	2 7 9 5 2	0 2 7 1 1	2 5 2 4 1
Marital status			
Single In a relationship Married Divorced	2 2 17 4	1 1 8 1	1 1 9 3
Number of children			
0 1 2 3 or more	8 4 10 3	4 2 4 1	4 2 6 2
Genetic group			
*C9orf72* *GRN* *MAPT* *TARDBP*	17 5 2 1	7 3 1 0	10 2 1 1
Education			
Low (ISCED^a^ 1–2) Intermediate (ISCED 3–4) High (ISCED 5–6) Higher (ISCED 7)	1 9 9 6	0 4 3 4	1 5 6 2

^a^ISCED: International Standard Classification of Education.

### Findings

**Fig. 1:**
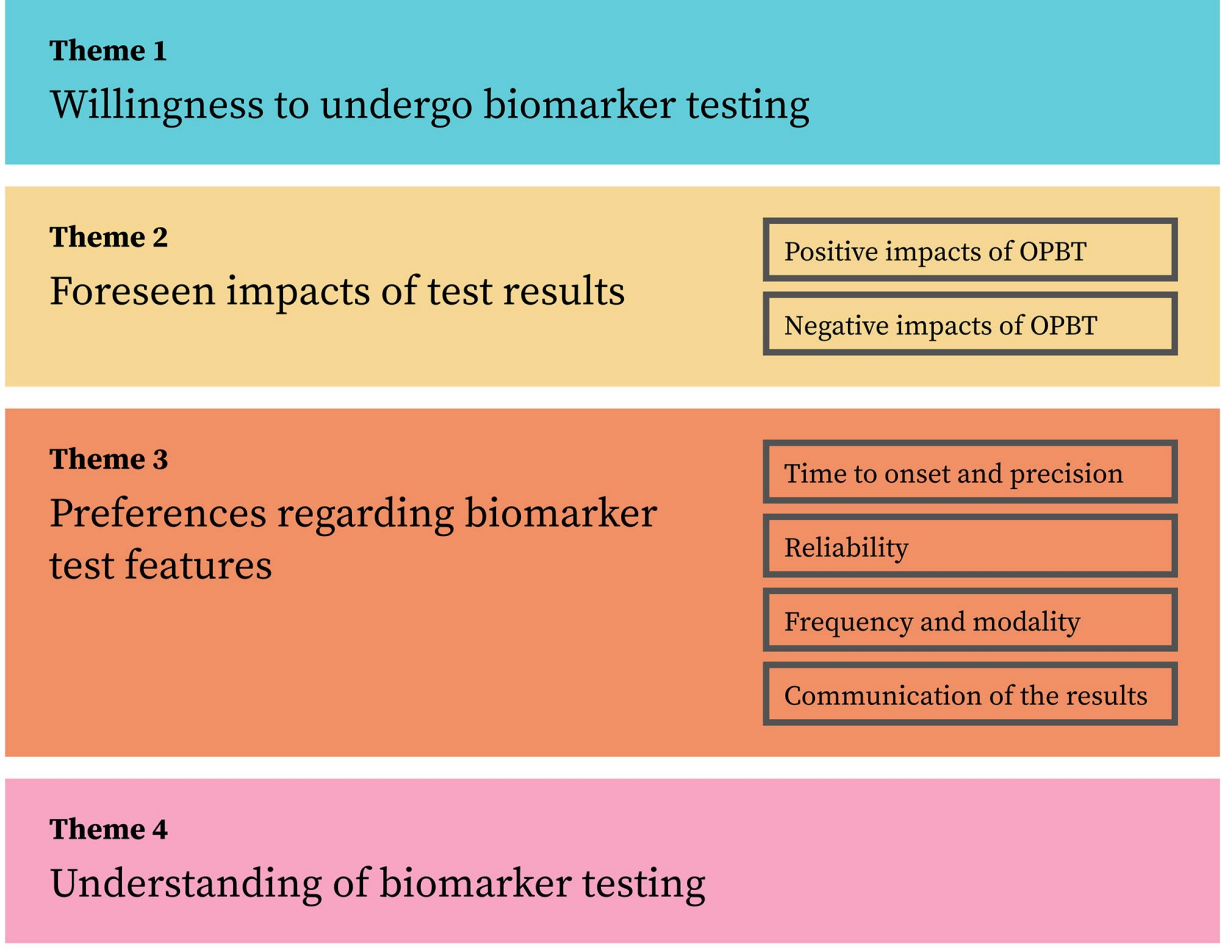
Overview of the themes and subthemes

Figure 1 shows the themes used to report the findings below. Most findings were similar in known mutation carriers and individuals at 50% risk. Any differences between these groups are explicitly stated. Quotes illustrating the results are presented in Tables 2, 3, 4, 5 and 6.

### Theme 1: willingness to undergo biomarker testing

Both known presymptomatic mutation carriers and individuals at 50% risk of developing FTD were willing, even eager, to undergo OPBT when available (Q1, Q2). Some participants were not entirely sure, and said they would have to decide at the moment the test becomes available. Sometimes age was an influencing factor, as participants would be more willing to undergo OPBT at age 50 or 60, when the risk of onset being imminent was perceived as higher. At the same time, they expected their willingness to decrease again at an older age (from 70 onwards). At that age, they would expect disease onset to occur anyway and a positive OPBT result would not provide new information. Participants did not object to disclosure of OPBT results as part of an invitation to participate in a clinical trial. They were generally highly motivated to participate in clinical trials, either in the hope that the experimental intervention might work, or to contribute to the development of interventions for next generations of patients (Q3).

In individuals at 50% risk, willingness to learn their genetic status in order to be included in OPBT monitoring for clinical trial eligibility varied. Some were only willing to participate in OPBT if their genetic carrier status would be disclosed only after OPBT results turned out positive (Q4). Other participants at 50% risk did not object to learning their genetic status before OPBT monitoring, if this were a condition.

Some participants would only consider OPBT if a clinical trial for an experimental intervention was available (Q5), but most participants stated they would also want to undergo OPBT if no (experimental) intervention was available. The reason for this was that they saw many positive impacts from OPBT apart from clinical trial participation (see Theme 2). One participant at 50% risk was not willing to undergo OPBT under any condition, because he thought he would not be able to handle the psychological impact of a positive OPBT result (Q6).

**Table 2:** Quotes illustrating willingness to undergo biomarker testing

	Participant ID, genetic status, gender, age	Quote
Q1	R9, known carrier, female, 40–50	“It seems enormously valuable to me if you [could] know in advance: you are going to become ill over the next few years.”
Q2	R6, 50% risk, male, 50–60	“[If I underwent genetic testing I would] know immediately, and then what can I do with it? So that is insufficient for me at this time. Or well, I just don’t want to know. But if I could do a [biomarker] test now and I would get it [FTD] in two years, then I *would* want to know that.”
Q3	R9, known carrier, female, 40–50	“[I would participate] because it teaches people something. And if it is not right for me, then it is useful for someone else in the future. Especially that is a beautiful motive to [participate in research] I think, because it probably will not help me anymore, but perhaps it will help the person in generations after me. So I think I would do it. Well, also for yourself of course, but the chances that it [works] for that, well…”
Q4	R3, 50% risk, female, 30–40	“I would rather first be monitored and only then have genetic testing, but mostly because I do not want that genetic test result immediately. (…) I would only want the genetic testing result at the moment that the blood [biomarker] test changes, that the idea is that it is starting.”
Q5	R5, known carrier, female, 40–50	“I would only want to know if I can do something to slow the disease process or delay it or, preferably, cure it, of course.”
Q6	R16, 50% risk, male, 20–30	“I would not be able to handle it, I think, no. [What makes it difficult is] that you know what is going to happen, because you know what the rest of your friends and family… You will not notice yourself (…) The rest will be broken by it, sort of, especially your loved ones.”

### Theme 2: foreseen impacts of test results

#### Positive impacts of OPBT

Both known mutation carriers and individuals at 50% risk felt that OPBT could provide them with valuable information about the near future. A positive OPBT result would allow them and their family the opportunity to mentally prepare for onset and make changes in the remaining healthy years. Almost all participants stated that a positive result would lead them to enjoy life more, fulfil life goals sooner and spend more time with their loved ones in their final healthy years. Life goals often amounted to travelling or spending more time on hobbies (Q7). Participants foresaw that a positive OPBT result would lead them to reduce their work hours or retire, to avoid FTD affecting the quality of their work and also to spend more time doing the things they love. In addition, a positive OPBT result might lead to discussions at work about the implications of FTD for their role, finishing up their work well, or possible adaptations to their position (Q8). These life planning intentions did not appear to differ between confirmed mutation carriers and individuals at 50% risk.

Participants also said that they would start planning to reduce the impact of FTD on their family as much as possible. This was partly inspired by the impact they had experienced in caring for their parent with FTD. A positive OPBT result would lead them to draw up a will and plan for future care (e.g. early admission to a home, to prevent their family from carrying the burden of care) and the continuation of family life without them (e.g. concerning mortgage payments and life insurance). In addition, receiving a positive OPBT result would be the right moment to draw up an advance care directive or re-discuss possibilities for the end of life and euthanasia with their general practitioner and loved ones. Participants expressed the concern that they might not be able to receive euthanasia before they become mentally incompetent to make decisions. Therefore, some participants mentioned that they might try to request euthanasia early in the disease process to avoid being “too late” (Q9). One participant mentioned that she would consider committing suicide to avoid burdening her doctors with the process of euthanasia. The intentions to draw up advance care directives and explore options for the end of life were similar in confirmed mutation carriers and individuals at 50% risk.

Participants mentioned that a positive test result would have an effect on their lifestyle. For some, the effect would be that they would take up a healthier lifestyle or turn to alternative medicine, hoping to delay symptom onset in that way. Other participants said that they would pay less attention to their general health, e.g. by spending less time working out or by adopting a more abundant diet, since imminent symptom onset would reduce their life expectancy anyway (Q10).

Besides impacts from receiving a positive OPBT result, participants also commented on foreseen impacts of a negative result. Participants said a negative result would lead to relief that symptom onset is not expected soon, even though this relief would be temporary (Q11). In general, participants stated that they would not make any life changes after receiving a negative result.

**Table 3:** Quotes illustrating positive impacts of OPBT

	Participant ID, genetic status, gender, age	Quote
Q7	R15, 50% risk, female, 50–60	“Reduce stress, do good things for myself, good diet, yes, spend more time with my son. (…) But yeah, not postponing anything anymore, going on trips, do nice things.”
Q8	R11, 50% risk, male, 40–50	“I work five days a week now. Well, would you still do that then? Or would you say: hang on, yes, I am going to phase out, work fewer days, but do other things that I enjoy. (…) Of course you have to look at your role, like: how can you keep functioning in your role for example? (…) I think you can still be of good value for a very long time without being troubled by the dementia part, actually.”
Q9	R14, known carrier, female, 50–60	“In the ideal situation, I say at the moment that I am still competent: I am just stepping out [ending my life]. And I hope that I will be brave enough for that at that time. It’s not about living as long as possible for me.”
Q10	R19, 50% risk, female, 30–40	“Maybe that I become more relaxed [with my health] then. (…) Then it does not matter anymore, because then I cannot slow it down anymore. Right now I still just think about my general health (…) then it matters less.”
Q11	R23, known carrier, female, 30–40	“A sort of relief, like: it’s not there yet. I can breathe for a while. But then maybe also that the question pops up, like: well, when, then, with the next [testing moment]? What will be the result then?”

#### Negative impacts of OPBT

Participants also mentioned disadvantages of OPBT. The psychological burden of receiving a positive OPBT result was mentioned most frequently. Multiple participants said it would feel like “being slapped in the face”, while others used words like “shocked”, “emotional”, “broken”, “panicking”, “losing hope”, “dejected”, “heavy”, “relieved” (to be aware sooner rather than later), “lousy”, “sad”, “depressed”, “impacted”, “mentally exhausting” or “resigned” for potential psychological reactions they might have (Q12). However, participants usually also indicated that it was difficult to predict how they would react. Most participants estimated they would be able to cope with a positive result, but a few participants felt that the foreseen psychological impact on them and their loved ones might be reason enough not to undergo OPBT (Q6). A different psychological burden described by participants was the expected stress around repeated OPBT moments and disclosure. Both mutation carriers and individuals at 50% risk mentioned it. Stress was viewed as a potential disadvantage of repeatedly undergoing OPBT over the years as part of monitoring for clinical trial eligibility (Q13).

Besides these psychological burdens of OPBT, participants foresaw two other negative impacts from OPBT. Firstly, some participants expected that a positive OPBT result might suddenly affect their self-image or other people’s image of them. They might start to act in line with the OPBT result, or other people might treat them differently when hearing about the positive result (Q14). Consequently, participants would only tell specific people about the result, like (close) family, (close) friends and people who would be affected by their having FTD. In a similar vein, one participant mentioned that she would wait with OPBT until she had settled her mortgage, as she feared that receiving a positive OPBT result might affect that. Secondly, even though OPBT results would provide more information on expected time of onset, some uncertainty would remain regarding the pace of progression and the nature of the symptoms to be expected at the estimated time of onset (Q15).

Despite these disadvantages and potential negative impacts of OPBT, most participants stated they would still be willing to test, because the perceived usefulness of OPBT outweighed the disadvantages.

**Table 4:** Quotes illustrating negative impacts of OPBT

	Participant ID, genetic status, gender, age	Quote
Q12	R23, known carrier, female, 30–40	“Maybe I will be shocked, for a moment become emotional from the fact that judgement day is coming. But on the other hand I imagine that I will be able to turn it around into: well, let it come then. Because I’ve known for years that I am a carrier and that the risk is that it is coming. So it is not a bolt from the blue, you know?”
Q13	R6, 50% risk, male, 50–60	“And that [you think]: oh, well, we have the test again this year, [that causes] tension. With some people, it gets in their system, perhaps not me, but I think with [my partner] it will, that she is occupied by it, unconsciously, like: fingers crossed, let’s hope that [the result is normal].”
Q14	R10, known carrier, female, 40–50	“Because then everything that you perhaps accidentally do differently than normally, then you are immediately being watched [by others]. So I expect that that will happen then. Yes, it is also a bit that then you suddenly become a patient, you know, because you suddenly have a disease.”
Q15	R10, known carrier, female, 40–50	“It says something, but it does not say a lot, because then the symptoms start. Well, then that could be in the coming, one, two or three years, but then you also don’t know how quick or slow it goes. (…) Because then you also want to know: well, what will I notice? And you don’t know that either.”

### Theme 3: preferences regarding biomarker test features

Participants were asked about their preferences regarding modality, frequency and predictive value of OPBT, as well as the communication of the result.

#### Time to onset and precision

The preferred time from the moment of receiving a positive OPBT result to the onset of symptoms (“time to onset”) was two to five years. If it were shorter, such as a year, some felt that this would not leave enough time to prepare. Others did not mind such a short time to onset, because any warning of coming symptoms would be appreciated. If time to onset were up to ten years, participants thought receiving a positive result so far in advance would diminish the value of the information. Such a test result was sometimes equated with presymptomatic genetic testing, especially by participants in their 40s or early 50s. These participants expected the disease to start sometime in the next ten to twenty years anyway (should they be mutation carriers). A few participants did not mind a time to onset of ten years or longer or even preferred it, as long as the estimate were precise. In this way, the result would mean that eight to nine years of good health could still be expected. More generally, almost all participants preferred more precise estimates, preferably within the range of a year. They usually felt that more precise information would lend itself better for planning and making changes (Q16).

#### Accuracy

Participants understood that OPBT would not be 100% accurate, but considered a high accuracy of both positive and negative OPBT results important to avoid making radical choices based on false results (Q17). Participants stated that OPBT results should be correct in at least 90 to 95 out of 100 people (see Supplement 2 for formulation of question). Others also accepted a predictive value of approximately 70 out of 100. Usually, their preference for the positive and negative predictive value was the same. In any case, participants made clear that the accuracy of the test should be communicated by the healthcare professional at the moment OPBT was offered, so that they could make an informed choice whether to test or not.

#### Frequency and modality

The frequency and modality of OPBT generally did not affect willingness to test. All participants stated a preference for testing in blood or using an MRI scan rather than a lumbar puncture. However, most participants would still do the test if it required a lumbar puncture or if that modality provided a more precise or accurate result.

Participants often stated that they would expect a testing frequency of once every year or every two years. In general, very frequent testing (e.g. monthly) was considered undesirable, due to the potential stress surrounding testing moments (Q18).

#### Communication of the results

Preferences for communication of the test result differed, with the majority of participants preferring disclosure in person. Others preferred a video call or telephone call. Almost all participants would like at least some form of real-time communication, because this felt more personal to them and allowed for the opportunity to ask questions directly. A few participants said that an e-mail or a letter would be acceptable to them.

Preferences also varied regarding whether or not the moment of disclosure should be agreed upon in advance, and whether negative results should be communicated explicitly. For example, one participant mentioned that she preferred to be notified only in case of positive results and not to actively receive negative results, as this would increase the tension of being tested.

**Table 5:** Quotes illustrating preferences regarding OPBT features

	Participant ID, genetic status, gender, age	Quote
Q16	R3, 50% risk, female, 30–40	“I think ten years is very long. The longer the period, the closer you get to testing yourself for the gene, because I could test myself for the gene now and then I have the risk that I become ill in 25 years. That is a pretty long period, and then I think: well, what can I do with that knowledge at this time? I think when the period is a bit shorter, then the need to do something with that information becomes bigger.”
Q17	R17, known carrier, male, 40–50	“I’ll give an example. If I say to someone: ‘You have a year left.’ And he spends all his money in one year, and then you say: ‘Well, sorry sir, but you have another four years.’ (…) If you say to someone for example: ‘You have a year left’, he will live toward that moment the whole year. And when it turns out after a year that nothing is going on… it has to be accurate.”
Q18	R18, 50% risk, female, 20–30	“It’s also not that it should become a part of my life, that every month I [think]: oh right, here I go again. So no, I think one, two times every year, that I would find that sufficient. You know, otherwise I think you are too preoccupied with it, like: is it coming, is it coming?”

### Theme 4: Understanding of biomarker testing

When discussing implications of OPBT results, participants generally appeared to understand the nature of the test well, independent of their level of education. However, sometimes their presuppositions about what it could and could not predict differed. Firstly, some participants did not view the OPBT result as binary (elevated or normal, terms used by the interviewer) but rather as gradual. Some also said that finding elevated results over time would increase the reliability or precision of the test result, or that the degree of elevation might be correlated with the severity of symptoms (Q19, Q20).

Participants also had varying views about the impact of a positive test result on their perceived health status. One *C9orf72* carrier and one *GRN* carrier felt like they had been diseased all their life, with the positive OPBT result revealing an acceleration of this process of being or becoming ill (Q21). Other participants thought a positive result would signal the start of the illness, suddenly making them ‘a patient’ (Q22). Still others saw a positive test result as a prediction that one is due to become ill soon, when symptoms start, although these symptoms might not be immediately recognised (Q23).

**Table 6:** Quotes illustrating Understanding of OPBT

	Participant ID, genetic status, gender, age	Quote
Q19	R7, 50% risk, male, 50–60	“I am assuming that if you doubt [the result] (…), you could measure again, then you could probably deduce whether it has become worse or not. Depending on how you test of course, it could be that the test just says: yes or no, but I am assuming that there is a certain degree or value in it.”
Q20	R1, known carrier, female, 30–40	“I think it would be nice to get tested more often, so that you also know exactly how it is developing and how bad you can expect the symptoms to get. I think that those blood tests can tell you better than that it is visible from the person.”
Q21	R8, known carrier, female, 40–50	“Look, I have a mutation and so I have been making less protein than you all my life (…) Yes, but from the moment of conception I have been becoming ill, only it [the symptoms] still just has to happen.”
Q22	R25, 50% risk, female, 40–50	“I would become very insecure. Yes, I would then feel that I am deteriorating (…) if it [the OPBT result] is beyond the normal range, then I would feel ill.”
Q23	R24, 50% risk, male, 30–40	“[Receiving a positive OPBT result] is a different conversation than: ‘We see symptoms now and it has started’ (…) It’s the difference between ‘reckoning with’ and ‘you are ill’.”

## Discussion

Key findings of this study are that the large majority of participants at risk of developing genetic FTD in this study was willing to receive OPBT results in the context of clinical trial recruitment. This included individuals at 50% risk who were not willing to learn their genetic status at that time. Our results suggest that an important reason to undergo OPBT was the perceived actionability of results, as these make timely changes in life planning possible. Participants envisaged that a positive OPBT result would impact a wide range of personal and social domains: how much they work, how they spend their time, whether and how they prepare for future care and the end of life, how they and their actions are perceived by those around them, and how they experience life itself. For individuals at 50% risk, the perceived actionability of OPBT results stood in contrast to presymptomatic genetic testing results. They felt that the information about time to onset provided by OPBT would be more reasonable grounds for change than genetic information alone. Still, the value of OPBT lay not only in the actionability to plan life, but also in the impact it might have on one’s attitude in life. Positive OPBT results would urge one to spend the remaining time before onset well and enjoy life.

When comparing the high willingness to undergo OPBT found in this study with the existing literature, the results appear to be largely in line with those found in previous studies. A British survey study found that 85% of 135 survey participants at risk of FTD would be willing to receive NfL results as part of clinical trial recruitment [17]. Only 34% of the study population were known mutation carriers, so this cohort also contained many people at 50% risk who would be willing to learn NfL results in the context of clinical trial recruitment. Two survey studies in autosomal dominant Alzheimer’s disease (ADAD), for which the mutation is highly predictive of the age of onset [21], showed that persons at 50% risk of being a carrier may be willing to undergo genetic testing if they could subsequently participate in a clinical trial, with one study reporting 72% of at-risk persons to be interested in genetic testing for this purpose [22, 23]. However, participants at 50% risk in our interview study perceived value from OPBT results *independent of* their ability to participate in a clinical trial. This is in contrast with the low willingness (around 10%) to learn their genetic status among individuals at risk of ADAD outside the context of clinical trials [24]. One relevant difference between the two groups may be that carriers of ADAD mutations would receive an estimate of age of onset perhaps decades in advance, while individuals at risk of FTD would receive a prediction of symptom onset in the next few years. Participants at 50% risk in our study thought the short time to onset made the information more actionable, increasing its value and their willingness to learn their genetic status.

Moreover, participants in this study appear to foresee fewer harms from disclosure of OPBT results than researchers do, and expect that they would be able to cope with the psychological burden of receiving positive OPBT results. Existing literature on the psychological impact of learning apolipoprotein E *(APOE)* status or amyloid status in the context of Alzheimer’s disease (AD) suggests that negative psychological consequences are rare [25–28]. Yet, these results cannot be directly extrapolated to OPBT in genetic FTD. From what is currently known, a positive amyloid PET result at age 65 in cognitively healthy persons is associated with a 21.9% lifetime risk of developing AD dementia for men and a 29.3% lifetime risk for women, compared to 19.5% and 21.1% respectively in the general population [29–30]. Being homozygous for the *APOE* ε4-allele is associated with a 48.3% risk of developing AD by age 85 [31]. This makes the information provided by OPBT in FTD – if indeed it proves to be of sufficient clinical validity – qualitatively different from the information provided by AD biomarkers, in at least three ways. Firstly, amyloid PET results and *APOE* status indicate an increased lifetime risk of disease, and amyloid buildup may be present as long as fifteen years before symptom onset, while OPBT results provide an estimation of time to onset of a few years. Secondly, most individuals with an increased risk of AD will develop dementia at a later age than individuals at risk of early-onset FTD. Thirdly, cognitively unimpaired members of the general public are not already living with a Sword of Damocles at baseline, as (potential) mutation carriers are, due to their high genetic risk. These differences between amyloid and *APOE* testing for AD in cognitively unimpaired research participants and OPBT in (potential) carriers of genetic FTD may lead to differences in psychological impacts. In practice, the immediacy of a positive OPBT result in FTD – as opposed to the long-term potential of developing AD dementia with positive amyloid results – might increase the negative psychological impacts of OPBT results. For example, positive OPBT results might burden individuals at risk of FTD with approaching symptom onset, cause anxiety or dejectedness about the future, and might cause them to feel like a ‘patient’ already, increasing hypervigilance. Alternatively, they might lead to abnormal behaviours being (unduly) perceived as symptoms more quickly. This might change the experience of disease onset. At the same time, individuals at risk of FTD may welcome the certainty about onset that OPBT results provide compared to their current uncertainty about age of onset – again, if they prove to be of sufficient clinical validity. Overall, the psychological effects of receiving OPBT results in genetic FTD are unclear and need to be elucidated in empirical studies, if disclosure of OPBT results is implemented. We recommend that longitudinal studies are conducted to assess psychological outcomes of disclosure of OPBT results, including measures of anxiety, depression, quality of life, disease perceptions and coping mechanisms at different time points after disclosure, and assessments of impacts of disclosure on family members.

This study has implications for clinical trial recruitment in FTD. It found that some individuals at 50% risk may be willing to undergo OPBT only if they are not required to undergo presymptomatic genetic testing first. Currently, there is a presumption in favour of only offering OPBT for clinical trial recruitment to known mutation carriers. The current condition of undergoing genetic testing before undergoing OPBT is meant to protect individuals at 50% risk against learning two highly emotional and potentially harmful pieces of information at the same time, namely that they will develop FTD *and* that they will develop it soon. However, the positive impacts of OPBT foreseen by participants in this qualitative study suggest that these potential harms may be outweighed by the advantages of undergoing OPBT, and that individuals at 50% risk also wish to be invited for clinical trial recruitment using OPBT.

Finally, this study underscores that there is an urgent need for ethical guidance for clinicians and researchers on how and to whom to offer OPBT in the context of clinical trial recruitment. This is especially important if participants in cohort studies like FTD-RisC [32], the Genetic Frontotemporal dementia Initiative (GENFI) [33] and the ARTFL LEFFTDS Longitudinal Frontotemporal Dementia (ALLFTD) study [34] will be offered OPBT at regular intervals in the future. It is possible that OPBT may have larger impact than presymptomatic genetic testing, since it predicts time to onset rather than the risk of developing FTD at some point in life. Participants in our study expressed the wish to receive adequate counselling if OPBT were offered to them. This would support them through the process of decision-making and coping with the result, as is currently the standard for presymptomatic genetic testing in neurodegenerative diseases. Traditionally, the standard for genetic counselling includes: (a) a pre-test information session about features of the genetic test and reasons for and against testing, (b) a waiting time to allow sufficient reflection, (c) a post-test disclosure session in which the implications of the result are discussed, and (d) a protocol for follow-up to ensure sufficient (psychological) support to process the genetic test result [35, 36]. A similar counselling process might be appropriate for OPBT in the context of longitudinal cohort studies, to meet the needs of participants, support their decision-making, and help them deal with the results. The provision of psychosocial follow-up care is especially important, and guidelines should include suggestions for the management of individuals who experience decisional regret after disclosure. Future studies could focus on developing such a format for OPBT counselling in the context of longitudinal cohort studies. It could be based on existing counselling guidelines and tailored to the preferences of individuals at risk of genetic FTD.

### Strengths and limitations

A limitation of this study is that it has included only Dutch participants, while culture and national healthcare context may significantly influence willingness and perceived value and harms of OPBT. Firstly, Dutch culture highly values self-direction, including personal autonomy [37]. Individuals at risk of FTD in other countries may not value control over life planning in the same way, or may have more limited opportunities for early retirement or planning care in advance. Thus, they may not perceive OPBT results as actionable or relevant, potentially leading to a lower interest in learning this type of health information. Secondly, euthanasia in dementia patients is possible in the Netherlands, albeit bound to stringent due care criteria [38]. Individuals at risk of FTD in countries that prohibit euthanasia or do not endorse it as an acceptable practice, will thus not perceive this as a benefit of OPBT. Thirdly and more broadly, Dutch citizens generally have equal access to good-quality healthcare, as health insurance coverage is mandatory, government-supported, and includes long-term care [39]. Individuals in other countries may not have access to follow-up care. Besides cultural context, other factors, such as socioeconomic status, religious beliefs, and participant occupations, may also influence perspectives and decisions beyond education level. Overall, future research should involve participants from different cultural, socioeconomic, religious and occupational backgrounds, to see whether (potential) carriers’ perspectives differ across these factors. In addition, the potential cultural differences in reactions to OPBT results may also give reason to adapt guidelines to the cultural context in which they are implemented.

Still, this study provides rich and detailed data about Dutch (potential) carriers’ perspectives on OPBT from a sample diverse in gender, age, geographical location in the Netherlands, and education level. This increases the chances that the sample captured a large part of the range of perspectives on OPBT in the Netherlands. In addition, participants were recruited both from a research context and clinical practice at the Department of Clinical Genetics. This broadened the population to include people who are not active participants in research, and who may view tests and personal health information differently due to their lack of experience with observational research. We did not recruit participants who have not been in contact with healthcare professionals. However, since OPBT will in the foreseeable future only be offered in the research context, the considerations of individuals who are involved in research or at least in contact with healthcare professionals are most relevant.

A disadvantage of qualitative studies is that they do not provide quantitative estimates of the frequency of findings in the population, due to the limited number of participants. Further cohort-wide studies could examine what percentage of current research participants would be willing to undergo OPBT in the context of clinical trial recruitment. This could provide additional information on the feasibility of using OPBT as an inclusion criterion for clinical trials. Researchers undertaking such a survey study should formulate survey questions carefully, as the formulation of the questions and the information provided about the test may influence the results.

In addition, the hypothetical nature of OPBT may have made it difficult for participants to envision under what conditions they would or would not undergo OPBT and how they would react to a positive result psychologically. It may be possible that the range of psychological impacts occurring if OPBT results are actually disclosed will be wider than those foreseen by this study’s participants. Furthermore, actual testing rates in the future may not match the willingness to test reported in this study. This was previously seen in presymptomatic genetic testing in Huntington’s disease, for which 65–79% of survey respondents had stated an intention to test before genetic testing was available, but only 12–17% actually did undergo testing [40–44]. Still, the current study used qualitative methods, which may more reliably indicate intention to test than survey studies would. Once OPBT is offered in practice, research on the willingness to test and its psychological effects will need to assess whether the results of this study reflect actual willingness and psychological effects. In addition, repeat interviews may indicate whether individuals’ willingness to undergo OPBT is stable or changes over time.

## Conclusions

This study shows that Dutch known presymptomatic mutation carriers and individuals at 50% risk of FTD are willing to receive OPBT results in the context of clinical trial recruitment. In their eyes, the expected advantages of receiving OPBT results outweigh the expected disadvantages. These results provide important insights for clinical researchers and Institutional Review Boards to consider in the development of ethical guidance for informed consent, disclosure and clinical trial recruitment in the field of FTD research. Such guidance should balance the tension between the perceived value of OPBT in the eyes of known mutation carriers and individuals at 50% risk, the uncertain predictive value of current OPBT methods, and the potential harms from disclosing OPBT results and genetic status, while considering potential cultural differences between populations. In addition, the results of this study could serve as input for clinical guideline developers and clinicians to determine under what conditions OPBT could be translated to the clinical context in the future.

## Electronic supplementary material

Below is the link to the electronic supplementary material.

Supplementary Material 1

Supplementary Material 2

Supplementary Material 3

## Data Availability

The datasets generated and/or analyzed during the current study are not publicly available to protect the privacy of participants with rare genetic risk for FTD. The datasets are available from the corresponding author on reasonable request.

## Abbreviations

AD: Alzheimer’s disease
ADAD: Autosomal dominant Alzheimer’s disease
ALLFTD: ARTFL LEFFTDS Longitudinal Frontotemporal Dementia study
CHG: Charlotte Graafland
EMB: Eline Bunnik
Erasmus MC: Erasmus Medical Center, University Medical Centre Rotterdam, the Netherlands
FTD: Frontotemporal dementia
GENFI: Genetic Frontotemporal Dementia Initiative
ISCED: International Standard Classification of Education
NfL: Neurofilament light chain
OPBT: Onset-predictive biomarker test
