# Supplementary material for: “It seems enormously valuable to me.” Perspectives of Dutch (potential) carriers of genetic FTD on onset-predictive biomarker testing
Source: Alzheimers Res Ther. 2025 May 6;17:99. doi: 10.1186/s13195-025-01749-z (PMC12054206; doi:10.1186/s13195-025-01749-z)
Supplement: Supplementary file 1 — Supplementary Material 1 [file 13195_2025_1749_MOESM1_ESM.docx]

Supplement 2. Interview guide

Underlined questions were the focus of the interview. Text in italics was information provided by the researcher.

**Topic 1: Process of discovering genetic variant for FTD in the family (to warm up)**

When was it first suspected that there could be a hereditary form of FTD in the family? How old were you then?

How long did it take before the presence of a mutation for FTD in the family was confirmed and certain?

What impact did it have on you to learn about genetic FTD in the family? Did your interpretation of the past and the behavior of your parent change?

**Topic 2: Genetic testing**

Did you choose to undergo genetic testing or not? How long did it take for you to make that decision?

*For persons who tested:* when did you test, and how old were you? How long did it take after the discovery of the genetic variant in the family to get tested?

What were your reasons to (not) undergo genetic testing?

How much did the (choices of) family members influence your choice?

How satisfied are you with your decision (not) to test?

What would have been a reason for you to make a different choice regarding genetic testing?

*For persons who tested:*  How did you tell family members about your genetic testing result?

*For persons who are carrier:* How do you perceive carriership? Does it set you apart from other people, or did it influence your self-image?

*For persons who tested:* Did the genetic testing result play a role in life choices (partner, children, work, insurance), and if so, in what way?

*For persons who tested:* to what extent are you still uncertain about the disease? (onset, types of symptoms and FTD, progression) How does that uncertainty affect you?

What do you perceive as the onset of disease, has it started at diagnosis, or when you experience symptoms, or before then?

At what age do you expect the disease to probably start?

**Topic 3: Biomarker/ blood test [thought experiment]**

*In the future, it may be possible to undergo a blood test that predicts whether the symptoms of the disease will manifest in the coming years. In practice, this means that the disease is expected to start soon when the blood test gives an abnormal result. Imagine: researchers can use such a blood test to determine whether the disease will start within three years.*

*For persons who tested genetically:* Would you undergo such a blood test? Why (not)?

*A blood test could be offered to people who know that they are carriers, but also to people who do not (yet) know whether they are carriers. Individuals at 50% risk may be screened periodically, and when an abnormal result is found, they find out that the disease is expected to start soon. At the same time, they learn that they are carriers.*

*For persons who did not test genetically:* You chose not to undergo genetic testing. Would you be willing to get tested with this blood test? What factors influence your choice to test with this blood test or not in the future?

Would you want to periodically undergo this blood test? Why (not)?

What would you say is the meaning of an abnormal result for your health status? When receiving an abnormal result, would you see yourself as ill, or becoming ill, or not yet ill, or something else?

What would be the advantages of a blood test that can predict the onset of disease? And the disadvantages?

What would be the impact of an abnormal result in the following demains? And of a normal result (onset not expected soon)?

- Psychologically
- Effect on loved ones and friends
- Life choices (also care)
- Professionally and financially
- Family planning

With whom would you share the blood test result, and with whom would you not? Why?

Would you undergo the blood test if you were 10 years older? Or 10 years younger?

*Researchers would like to know what the optimal characteristics of a such a blood test should be.*

How precise should such a blood test result predict the onset of disease? For example, that symptoms start between now and two years, or that they start between 1.5 and 2 years from now? Should the test – as a manner of speaking – predict the day that symptoms start, or should it predict symptoms in a period of six months or a year? What do you prefer?

How far in advance should such a blood test predict the onset of disease? Six months in advance? 1 year? 2 years? What if it would be 5 years or 10 years?

How often would you want to undergo the blood test? (once every 1, 2, 5, 10 years?)

So far we have talked about a blood test, but there may be other ways to do such a test, like a lumbar puncture or a scan. Would it matter to you what form the test takes? What would you prefer?

Every test makes mistakes sometimes. That can mean that some people who get an abnormal result do not develop symptoms in the next three years, or that persons who get a normal result do develop symptoms in three years. How accurate should the test result be for you to consider acting on the result? If a 100 people get an abnormal result, how many should indeed get symptoms in the next few years? And with a normal result? [use pictograph if necessary]

In what way should the test results be communicated? (in a face-to-face consult, telephone call, letter)

**Topic 4: Blood tests and clinical trials**

*If researchers want to do a clinical trial to test a newly developed drug to see i fit works, they are looking for people who do not have FTD symptoms yet, but are expected to develop them soon. The idea is that the drug will work best in people who do not have symptoms yet, to prevent symptoms from occurring.*

Would you be willing to participate in such a preventive clinical trial? With what motivation?

*In this type of clinical trials, researchers want to include people who are both a carrier of FTD, and have an abnormal blood test result.*

For carriers: Would you be willing to be monitored (i.e. undergo the blood test periodically) to see whether you become eligible for participation in a clinical trial at some point?

For individuals at 50% risk: *there are two ways for researchers to assess who is eligible to participate in the clinical trial. The first option is to screen known carriers of the genetic mutation for FTD regularly using the blood test, and include them in the study when they receive an abnormal result. That means that you would not be able to participate, since you do not know whether you are a carrier or not.*

Would that be a reason for you to undergo genetic testing, in order to be eligible for monitoring your eligibility for clinical trial participation using blood tests (with the risk that you may not (immediately) be able to participate after genetic testing)?

*A second option is that researchers perform the blood test regularly in individuals at 50% risk, and only ask to do the genetic test after the blood test has given an abnormal result. If you then turn out to be a carrier, you would probably be eligible for participation in the clinical trial. You would learn in the same moment that you are a carrier of FTD and that the disease will start in the coming years.*

Would you want to undergo the blood test regularly in order to be monitored for clinical trial eligibility without first learning your genetic status? Which of the two options would you prefer?

Would you also want to receive the result of the blood test if it is still unclear how far in advance, how precise, and how accurate the result is? (how well in predicts onset)

**Final questions**

Do you have any additions? What do you want to emphasize?
