# Supplementary material for: “It seems enormously valuable to me.” Perspectives of Dutch (potential) carriers of genetic FTD on onset-predictive biomarker testing
Source: Alzheimers Res Ther. 2025 May 6;17:99. doi: 10.1186/s13195-025-01749-z (PMC12054206; doi:10.1186/s13195-025-01749-z)
Supplement: Supplementary file 2 — Supplementary Material 2 [file 13195_2025_1749_MOESM2_ESM.docx]

Supplement 1. Extensive methods

*Sampling and recruitment*

In order to gain a detailed understanding of the value of OPBT, we performed semi-structured qualitative interviews with known mutations carriers and individuals at 50% risk of genetic FTD. Most had no previous experience with OPBT for FTD (one participant had received negative NfL results). We used purposive sampling, aiming for diversity in age, mutated gene, education and sex. Inclusion criteria were: a) being ≥18 years old, b) speaking Dutch, c) carrying or being at 50% risk for carrying an identified pathogenic autosomal dominant mutation for FTD.

Participants were recruited from two contexts: people who had sought genetic counseling for genetic testing for genetic FTD at the department of Clinical Genetics, or participants in the longitudinal research cohort FTD-RisC at the Erasmus MC. The FTD-RisC cohort study is part of the GENFI consortium and has been running for over ten years. It aims to describe the natural history of conversion and clinical presentation of carriers of familial FTD, by following those at risk for genetic FTD. Participants visit the Erasmus MC (bi)annually for extensive neuropsychological testing, blood sampling, an MRI scan and optionally a lumbar puncture. They are offered the option of learning their genetic status, but are not required to, as this might present a barrier to research participation for those unwilling to learn their status, and data of non-carriers can be used as controls. We invited both FTD-RisC participants who are known mutation carriers and individuals that have chosen not to learn their genetic status for the interview study.

Prospective participants were approached for participation by the FTD-RisC neurologist (HS) or clinical geneticist (LD). When interested, an appointment was made via telephone by CHG, a female PhD candidate trained in medical ethics and qualitative research. Her project concentrates on the ethics of disclosure of OPBT results in genetic FTD, of which this interview study is an important element.

*Interview format*

The interview guide was first drafted by CHG and EMB, and then discussed with the FTD-RisC team (neurologists, clinical geneticists, neuropsychologists, medical ethicists). It was then pilot-tested with colleagues of the Medical Ethics department to optimize formulation of the questions. The guide concentrated on the following topics: 1) family history of FTD, 2) considerations concerning PT, 3) perspectives on OPBTs, including foreseen impacts and willingness to test, 4) perspectives on use of OPBTs as clinical trial eligibility criteria. At the beginning of the interview, the interviewer introduced herself and the aim of the research. When introducing the working mechanism of the OPBT, the interviewer concentrated on features of NfL testing, namely a biomarker test in blood that yields either an elevated level (compared to control values), signaling imminent symptom onset, or a normal level.

Interviews were performed during the period of September 2023 to August 2024 by CHG at the participant’s home or at the Erasmus MC depending on participant preferences. In three interviews, the partner was present in the room, but did not actively participate. The interview guide was adapted during the period of interviewing to include new questions on topics that were identified during previous interviews. During interviews, the formulation of the questions was sometimes adapted to the participant’s level of understanding. Interviews were audio-recorded and transcribed verbatim using online transcription service Amberscript, after which transcripts were pseudonymized by removing any information that could potentially lead to identification of the participant. The interviewer made field notes detailing first impressions directly after each interview. More participants were invited until data saturation was reached, defined as finding no new information in the last three interviews.

*Data analysis*

Inductive thematic analysis of the interview transcripts was performed using NVivo R1(2020) software. CHG started open coding during the period of interviewing, in order to identify improvements for the interview guide and topics to ask for in subsequent interviews, and to determine the point of data saturation. EMB used an open coding approach independently for two transcripts. These initial codes were discussed and used by CHG and EMB to construct an initial codebook. After coding the first six transcripts, the codebook was discussed again and agreed upon by CHG and EMB, for the coding of further transcripts, with room for small changes should those be necessary. All transcripts were coded separately by CHG and EMB, JLP or another colleague of the department of Medical Ethics, and these codes were compared in regular meetings to determine the final coding of all transcripts and the final codebook. The study design and results are reported in accordance with COREQ guidelines (see Supplement 3). In the Results section, a ‘positive’ OPBT result refers to the prediction that symptom onset will occur soon, and a ‘negative’ result to the prediction that symptom onset is not expected yet.
